# The reliability of a subtype-determining questionnaire in efficient benign paroxysmal positional vertigo diagnosis in geriatrics

**DOI:** 10.3389/fnagi.2023.1209342

**Published:** 2023-06-20

**Authors:** Yichen Wan, Yingxuan Li, Jianjun Sun

**Affiliations:** Department of Otolaryngology-Head Neck Surgery, Peking University International Hospital, Beijing, China

**Keywords:** benign paroxysmal positional vertigo (BPPV), questionnaire, diagnosis, geriatrics, treatment

## Abstract

**Introduction:**

Benign paroxysmal positional vertigo (BPPV), the most common cause of dizziness, especially for older adults, exposes patients to the lethal risk of falling. However, the diagnosis of BPPV in this population can be more elusive as they present few characteristic symptoms. Therefore, we explored the application of a subtype-determining questionnaire in BPPV diagnosis among the geriatric population.

**Methods:**

Patients were assigned to the aware and unaware groups. In the aware group, the technician would directly test the suspected canal indicated by the questionnaire, whereas, in the unaware group, the technician performed the regular positional test. The diagnostic parameters of the questionnaire were examined.

**Results:**

The accuracy, sensitivity, and specificity of questions 1–3 for diagnosing BPPV were 75.8, 77.6, and 74.7%, respectively. Question 4 demonstrated an accuracy of 75.6% in ascertaining the BPPV subtype, question 5 showed an accuracy of 75.6% in determining the affected side, and question 6 yielded an accuracy of 87.5% in distinguishing canalithiasis or cupulolithiasis. Examination time was shorter in the aware group than that in the unaware group (*P* < 0.05). No difference was found between the two groups for treatment time (*P* = 0.153).

**Conclusion:**

This subtype-determining questionnaire is practical in daily use and capable of providing instructive information for an efficient diagnosis in geriatric patients with BPPV.

## 1. Introduction

Benign paroxysmal positional vertigo (BPPV) is the most frequent vestibular disease that exhibits a cumulative lifetime incidence of 10% (von Brevern et al., 2015). The 1-year prevalence of BPPV increases with age (von Brevern et al., 2007), accounting for approximately 30% of older adult patients with vertigo (Balatsouras et al., 2018). BPPV adversely affects the quality of life and interrupts daily activities (Parham and Kuchel, 2016). Moreover, for older patients, it increases the risk of falls which can be lethal to this population (Ganança et al., 2010). It is reported that approximately 8% of general patients with BPPV have received proper treatment (von Brevern et al., 2007). Diagnosis can become even more elusive for older adults who present few characteristic symptoms and a more protracted course (Parham and Kuchel, 2016). Therefore, a swift and accurate diagnosis could be significant for these patients. Besides the widely recognized diagnostic measures such as Dix–Hallpike and supine roll tests, some scholars invented questionnaires based on the nature of the key elements of BPPV and explored the validity of this new tool (Friedland et al., 2016; Lindell et al., 2018; van Dam et al., 2020). The emergence of questionnaires has provided new ideas for BPPV diagnosis in addition to the traditional methods with their feasible prediction power (Higashi-Shingai et al., 2011). Questionnaires also bear advantages with regard to patients with limited spinal motion, atypical nystagmus, post-surgery status, subjective BPPV, or recurrence (Higashi-Shingai et al., 2011; Kim et al., 2020). Based on Kim et al.’s (2020) study, our previous study demonstrated that applying an innovative subtype-determining questionnaire is practical and beneficial when encountering dizzy patients (Wan et al., 2023). However, after an extensive literature review, only one study that specifically addressed the usage of diagnostic BPPV questionnaires among the geriatric population was found (Lapenna et al., 2016). The main purpose of this study was to reduce the risk of missed diagnosis of BPPV rather than attempting to elicit precise information about each BPPV precipitation. Therefore, this study aimed to explore the significance of the subtype-determining questionnaire in guiding the diagnostic and treatment procedure in older adults with BPPV.

## 2. Materials and methods

This prospective single-blinded study was conducted at Peking University International Hospital from September 2022 to March 2023. After ruling out three patients diagnosed with multi-canal BPPV, the remaining 153 patients aged ≥65 years with vertigo as their chief complaint were recruited. Patients with the following conditions were excluded from our study: intracranial pathologies, severe cardiovascular disease, severe spinal lesions, post-surgery status, major head/ear trauma, severe obesity, cognitive/communicative impairment, inability to endure the procedure, and spontaneous nystagmus.

The questionnaire used in our study was originally introduced by Kim et al. (2020) and was slightly modified by the authors (Wan et al., 2023). The questionnaire not only screens BPPV but also predicts the affected canal. The validity of its usage among general Chinese patients has been demonstrated in our previous study (Wan et al., 2023). The first three questions screen patients with BPPV, and the following ones aim to determine the subtype.

Participants were divided into unaware and aware groups on a random basis, in which the technician was blinded or informed about the questionnaire results, respectively. In the unaware group, the technician performed the supine roll test; if negative, Dix–Hallpike test was carried out. Initially, the technician would pick up either side, depending alternatively on the order of patients’ presence. The consideration and validity of adopting this order were elaborated on in our previous study (Wan et al., 2023). In the aware group, the technician knew the result of the questionnaire and would test the suspected canal straightforwardly. If BPPV was denied by the first three questions, the technician performed the positional test based on the aforementioned order.

Diagnosis of BPPV was established based on typical nystagmus elicited by the positional test. Patients were treated using Epley, Lempert/Gufoni, and Yacovino maneuvers according to their respective subtypes. Time spent on diagnosis and treatment was recorded.

### 2.1. Statistical analysis

We calculated the accuracy, sensitivity, specificity, positive/negative predictive value, and positive/negative likelihood ratio. Mann–Whitney U test was used for continuous variables, whereas the chi-square or Fisher exact test was used for nominal variables. A *P*-value < 0.05 was considered statistically significant. Statistical analyses were performed using R version 4.2.0^1^.

## 3. Results

A total of 153 older adult patients were included in this study. No significant difference was found between the aware and unaware groups in terms of age, course, sex, and proportion of true BPPV. In the aware group, the examination time was shorter than that in the unaware group, with statistical significance (*P* = 0.001). In patients verified as BPPV using the positional test, examination time remained shorter in the aware group (*P* = 0.000); however, the same conclusion could not be established for the treatment time (*P* = 0.153) (Table 1).

**TABLE 1:** Questionnaire used in our study.

Questionnaire for BPPV diagnosis by Kim et al. (2020) and slightly modified by us
**Question 1** Do you have spinning or a whirling sensation of the surroundings or yourself?
**Question 2** Do you feel dizzy mostly when your head is moved?
**Question 3** Does the dizziness last <3 min?
**Question 4** Which positional change makes you feel more dizzy? Lying down or getting out of bed? Turning your head (or body) **while lying flat or on the pillow?**
**Question 5** Which makes you more dizzy? Turning your head to the right? Turning your head to the left?
**Question 6** How long does the dizziness induced by head turning last? <1 min >1 min

BPPV, Benign Paroxysmal Positional Vertigo. Bold letter is the modified parts according to Chinese language habits, it was “while lying down” originally in Dr. Kim’s questionnaire.

We also analyzed the similarities and discrepancies between the groups regarding whether the accurate prediction was achieved using the questionnaire (identical results in both sides and subtypes using the positional test) (Table 3). No statistical difference was found in age, sex, course, if informed or not, sidedness, subtype, and treatment time; however, the accurately diagnosed group showed shorter examination (*P* = 0.046) and treatment time (*P* = 0.035) than that in the inaccurate group (Table 2).

**TABLE 2:** Comparison between the aware group and the unaware group in the elderly.

	Aware (*n* = 79)	Unaware (*n* = 74)	*P*-value	Estimated difference (95% CI)	Total
**In general**					
Age, Median (Q1, Q3)	69.00 (67.00, 76.00)	71.00 (67.00, 75.00)	0.417		71.00 (67.00, 76.00)
Course, Median (Q1, Q3) (d)	7.00 (3.00, 28.00)	7.00 (2.00, 20.50)	0.830		7.00 (3.00, 24.50)
**Gender n (%)**					
Male	28 (35.40)	20 (27.00)	0.262		48 (31.4)
Female	51 (64.60)	54 (73.00)			105 (68.6)
**BPPV**					
Yes	34 (43.0)	24 (32.4)	0.177		58 (37.9)
No	45 (57.0)	50 (67.6)			95 (62.1)
Examination time, Median (Q1, Q3) (s)	75.00 (42.00, 109.00)	97.00 (85.25, 107.25)	0.001	−23.00 (−36.00 to −10.00)	
**In verified BPPV patients**					
**Side**					
Left	15 (60.0)	19 (57.6)	0.853		34 (58.6)
Right	10 (40.0)	14 (42.4)			24 (41.4)
**Subtype**					
Posterior/ anterior	23 (60.5)	11 (55.0)	0.685		34 (58.6)
Horizontal	15 (39.5)	9 (45.0)			24 (41.4)
Examination time, Median (Q1, Q3) (s)	42.00 (24.00, 83.25)	100.00 (87.75, 106.50)	0.000	−55.00 (−66.00 to −40.00)	
Treatment time, Median (Q1, Q3) (s)	141.50 (105.50, 206.50)	167.00 (128.25, 224.25)	0.153		

**TABLE 3:** Comparison between the accurately diagnosed with the inaccurately diagnosed groups in confirmed BPPV patients.

	Accurate (*n* = 29)	Inaccurate (*n* = 29)	*P*-value	Estimated difference (95% CI)
Age	68.00 (67.00, 74.00)	71.00 (67.00, 76.00)	0.924	
**Gender**				
Male	8 (27.6)	9 (31.0)	0.773	
Female	21 (72.4)	20 (69.0)		
Course (d)	7.00 (2.00, 13.00)	4.00 (2.00, 10.00)	0.583	
**Aware or not**				
Yes	17 (58.6)	17 (58.6)	1.000	
No	12 (41.4)	12 (41.4)		
**Side**				
Left	10 (34.5)	15 (51.7)	0.185	
Right	19 (65.5)	14 (48.3)		
**subtype**				
Posterior/ anterior	22 (75.9)	16 (55.2)	0.097	
Horizontal	7 (24.1)	13 (44.8)		
Examination time, Median (Q1, Q3) (s)	56.00 (29.00, 95.00)	94.00 (42.50, 107.50)	0.046	−17.00 (−44.00 to 0.00)
Treatment time, Median (Q1, Q3) (s)	132.00 (105.00, 187.00)	174.00 (127.00, 271.00)	0.035	−35.00 (−66.00 to −4.00)

### 3.1. BPPV screening: Questions 1–3

**FIGURE 1:**
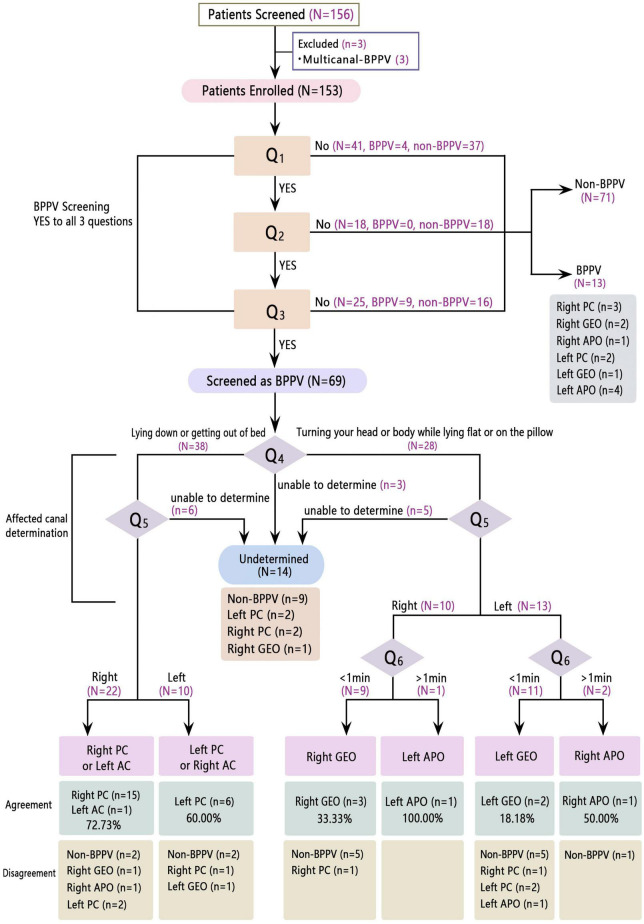
Flowchart of the algorithms and results of this subtype-determining questionnaire in geriatrics. PC, posterior canal; AC, anterior canal; GEO, geotropic; APO, apogeotropic.

As shown in Figure 1, among the 156 patients initially recruited who agreed to participate in this study, three were diagnosed with multi-canal BPPV and thus were excluded from further study. Of the 153 patients enrolled, 69 and 84 were assumed as with and without BPPV using the questionnaire, and the judgments were similar to the positional test results in 45 out of 69 patients and 71 out of 84 patients, respectively. Therefore, questions 1–3 could correctly decide whether a patient has BPPV in 116 out of 153 patients, with an accuracy of 75.8%. Other parameters, such as sensitivity, specificity, positive/negative predictive value, and positive/negative likelihood ratio were calculated using data shown in Figure 1 and demonstrated in Table 3.

### 3.2. Locating the culprit canal: Questions 4–6

Question 4 was designed to distinguish vertical canal from horizontal canal BPPV. Of the 33 patients verified having posterior/anterior canal BPPV using the positional test, 25 (75.8%) answered that they felt dizzier when lying down or getting out of bed (including three patients who answered the question as undetermined category, not shown in Figure 1, but was checked out from our data). Of the 12 patients confirmed having horizontal canal BPPV using the positional test (eight geotropic and four apogeotropic types), six (75.0%, including one patient with geotropic-type BPPV in the undetermined category, not shown in Figure 1, but was checked out from our data) out of eight patients with geotropic type and three (75.0%) out of four with apogeotropic type BPPV thought they felt dizzier when turning their body or head while lying flat or on the pillow. Hence the diagnostic accuracy of question 4 was 75.6% (34 of 45).

Question 5 was intended to determine the inflicted side of BPPV. Twenty-five out of 33 (75.8%) patients with posterior/anterior, seven out of eight (87.5%) patients with geotropic type, and two out of four (50.0%) with apogeotropic type BPPV chose the side consistent using the positional test. Therefore, the diagnostic accuracy of question 5 was 75.6% (34/45).

Question 6 was intended for patients who answered that they felt dizzier when turning their head or body while lying flat or on the pillow to determine the canalithiasis and cupulolithiasis. It had correctly predicted the subtype in 100% (5/5) of the patients with geotropic type and 66.7% (2/3) of those with apogeotropic type BPPV, yielding a diagnostic accuracy of 87.5% (7/8).

Among the 14 patients screened for BPPV but failed to give an assertive answer to either questions 4 and/or 5, nine (64.3%) were proved to not have BPPV.

## 4. Discussion

Benign paroxysmal positional vertigo is the most common diagnosis in dizzy patients, particularly older adults. Its 1-year prevalence increases sharply with age and can be seven times higher in the older population than in the younger population (Neuhauser et al., 2005). The prevalence of unrecognized geriatric BPPV is also high. In addition to the catastrophic consequence of falls rendered by BPPV, adverse psychological conditions, such as depression, disrupted daily activities, and avoidance of leaving the house can also be caused by this incapacitating disease (Parham and Kuchel, 2016). Older patients with BPPV report less rotatory dizziness, more unsteadiness, present with a more protracted course (Piker and Jacobson, 2014; Parham and Kuchel, 2016), and are often accompanied by various comorbidities. All these factors make BPPV diagnosis in this population more challenging. Currently, besides the classic positional test, scholars attempted to identify this disease by extracting the most representative elements about the nature of this disorder and integrating them into questionnaires to facilitate diagnosis (Higashi-Shingai et al., 2011; Friedland et al., 2016; Lindell et al., 2018; van Dam et al., 2020). Given the fact that presentation of BPPV is usually atypical among the geriatrics who report more unsteadiness and imbalance instead of the rotatory sensation because of their aging vestibular system, it just made us wonder if the subtype-determining questionnaire, the development of which was based upon the typical presentation of BPPV, still works as efficiently as it does among younger patients. Although few studies could be found on the application of a subtype-targeting BPPV diagnostic questionnaire in the geriatric population, we believe this field is worth looking into because of the potentiality of a reliable subtype-determining questionnaire in the swift identification of BPPV, making an efficient diagnosis, providing instructive information for otologist, and even guiding the patients’ home-based canalith repositioning procedure when medical service is inaccessible.

As shown in Table 4, the combined force of the questionnaire in diagnosing BPPV exhibited 75.8% accuracy, 77.6% sensitivity, and 74.7% specificity. Therefore, it is worthy of attention when compared with the data in our previous study of the general population (Wan et al., 2023), which showed an overall sensitivity of 90.9% and negative predictive value (NPV) of 93.9% for questions 1–3; these two parameters were significantly low in the geriatric population study (*P* = 0.008 for sensitivity; *P* = 0.008 for NPV). We believe this weakening in sensitivity and NPV power is well explainable and in concordance with the fact that older people tend to feel more unsteadiness or imbalance rather than rotatory vertigo, which phenomenon is essentially multisensory dizziness owing to the deterioration in the vestibular, proprioceptive, and central integration ability in the aging process (Balatsouras et al., 2018). The gradual vestibular loss enables older adults to process such change more properly and perceive more instability and movement intolerance, rather than the intense rotatory vertigo experienced by younger patients with BPPV (Fernández et al., 2015). Consequently, older patients without rotatory dizziness would be disapproved of having BPPV using the questionnaire, while typical nystagmus can still be evoked using a positional test that warrants a BPPV diagnosis. This may account for the relatively lower specificity and NPV of this questionnaire in the geriatric population. A similar result was presented by Lapenna et al. (2016) in their study of the anamnestic BPPV questionnaire in older adults. Nevertheless, with a 75.8% accuracy, 77.6% sensitivity, and 74.7% specificity of questions 1 to 3, we still consider this diagnostic property acceptable and applicable in daily practice.

**TABLE 4:** Diagnostic property of the questionnaire in the elderly.

	Accuracy (95% CI),%	Sensitivity (95% CI),%	Specificity (95% CI),%	Positive predictive value, (95% CI),%	Negative predictive value, (95% CI),%	Positive likelihood ratio, (95% CI),	Negative likelihood ratio, (95% CI),
Q1	59.5 (51.3, 67.3)	93.1 (83.3, 98.1)	38.9 (29.1, 49.5)	48.2 (38.7, 57.9)	90.2 (76.9, 97.3)	1.53 (1.28, 1.82)	0.18 (0.07, 0.47)
Q1 + Q2	71.2 (63.4, 78.3)	93.1 (83.3, 98.1)	57.9 (47.3, 68.0)	57.4 (46.8, 67.6)	93.2 (83.5, 98.1)	2.21 (1.73, 2.83)	0.12 (0.05, 0.31)
Q1 + Q2 + Q3	75.8 (68.2, 82.4)	77.6 (64.7, 87.5)	74.7 (64.8, 83.1)	65.2 (52.8, 76.3)	84.5 (75.0, 91.5)	3.07 (2.12, 4.46)	0.30 (0.18, 0.49)
Q4	75.6 (60.5, 87.1)						
Q5	75.6 (60.5, 87.1)						
Q6	87.5 (47.3, 99.7)						

The logic behind the designing of questions 4, 5, and 6 was elucidated in Kim’s work (Kim et al., 2020) and is easily understandable. Questions 4, 5, and 6 aimed at pinpointing the affected canal; therefore, only accuracy was calculable, which were 75.6, 75.6, and 87.5%, respectively. Surprisingly, the diagnostic accuracy of these three questions was similar to those in the general population, with 80.7% accuracy, 78.7% sensitivity, and 87.2% specificity (Wan et al., 2023). Originally, we expected less competent predictive ability of these parameters because hair cell and neuronal loss, a functional decline of the vestibular nerve, and reduced blood flow to the inner ear all contributed to an aging vestibular system (Zalewski, 2015; Ji and Zhai, 2018; Abdul Razzak et al., 2020). These degenerative changes happen in the forms of decreased cervical/ocular vestibular-evoked myogenic potentials amplitudes and abnormal head impulse (HIT) and modified Romberg tests (Davalos-Bichara and Agrawal, 2014; Fernández et al., 2015). This led us to assume a weaker power of questions 4 to 6. Nevertheless, the comparative diagnostic property of these three questions compelled us to look for an explanation. It is much easier to say it is due to the inclusion of older adults in the general population (different patient entity from this study); however, with only 21.1% (108/512) of patients aged ≥65 years in our last study (Wan et al., 2023), we believe this result deserves a deeper investigation.

The enhanced multisensory integration in the elderly seems to be a good candidate for an explanation, with the potential mechanism such as increased time window of integration; deficits in top-down attention control enable more distraction by stimuli through sensory modalities, inverse effectiveness, and elevated background sensory processing baseline (McGovern et al., 2014; Abdul Razzak et al., 2020). Similarly, Anson et al. (2016) found that older adults exhibited significantly larger compensatory saccades relative to the young in the HIT test, which was used to evaluate the function of the three semicircular canals. The intricacy of the vestibular system was recognized by virtue of its complex anatomy and sophisticated multi-integrational role in postural stability (Zalewski, 2015; Ji and Zhai, 2018). In fact, tests sometimes do fall short in detecting vestibular deterioration in aging on account of central compensatory mechanisms (Zalewski, 2015). In such context, it seems convincing that, as strong a stimulus as the otolith fell and moved in the semicircular canal, it was capable of irritating the vestibular system, which was, despite age-related degeneration, still functioning enough to perceive the stimulus by the provocative position in the right sense, credited to central compensatory mechanisms. We consider this might be accountable for a similarly good quality of diagnostic accuracy of questions 4 to 6 in comparison with the general population.

We noticed in Figure 1 that there were 36 patients judged by the questionnaire as having posterior/anterior canal BPPV, and only four showed negative results after the positional test; while this proportion is 10/30 in the questionnaire judged canalithiasis, a statistical difference existed (*P* = 0.028). This is in accordance with studies that spontaneous resolution is more frequent in the horizontal canal because the horizontal canal otolith has the predisposition to flow back into the utricle spontaneously by random head movement given the 30° tilt (Moon et al., 2006; Sekine et al., 2006; Pollak et al., 2018). Nonetheless, a multicentered descriptive study conducted in Spain didn’t find any difference of spontaneous resolve rate between different canal types (de Sande et al., 2019).

Examination time in the aware group was significantly shorter than that in the unaware group (*P* = 0.000), both in the general participants and those with verified BPPV. This finding forcefully endorses the value of this subtype-determining questionnaire in identifying the culprit canal and its practical value in aiding in the diagnostic process. An efficient diagnosis spares older adults unpleasant experiences of undergoing the positional test. Treatment time did not differ between the aware and unaware groups (*P* = 0.153), which is comprehensible due to the same repositioning maneuver adopted for each BPPV subtype.

Both examination and treatment time in the accurately diagnosed group was shorter than that in the inaccurately diagnosed group (*P* = 0.046), which is an interesting result that we propose the “optimal BPPV” theory for an explanation. Gradual aggregation of micro-otoconia over a long time period was advocated as a pathological change underlying BPPV precipitation (Ichijo, 2017). In addition, the movement of free otoconia in the semicircular canal may be affected by the composition of fibrils and matrix within it, a higher proportion of which would cause more impediments to its movement (Bojrab and Schutt, 2018). Therefore, it is plausible to deduce that during the accumulative process of the otolith, there is a certain “optimal” section when the otolith is around the right size and composition, which enables an unhindered movement within the semicircular canal. We believe such otolith movement gives the patient a more unambiguous perception of the most evocative head position instead of an inexplicit feeling when the otolith is either too small to trigger a clear sense or subjective BPPV (Jung and Kim, 2016) or too large and sticky to move in a common manner. Treatment time was also shorter in the accurately diagnosed group than that in the inaccurately diagnosed group.

The same phenomenon was observed in Kim’s and the present study of the general population (Kim et al., 2020; Wan et al., 2023), which we think were no coincidental findings. A higher proportion of fibrils and matrix contributes to increasing repositioning difficulties (Bojrab and Schutt, 2018), and refractory BPPV might happen if there are multiple deposit sites, which would not only muddle a clear perception of BPPV burst but also complicate treatment as they would block the returning pathway of otoconia (Ichijo, 2017). Therefore, conditions interfering with diagnosis might also make treatment more challenging. Therefore, these considerations might give us some hint in understanding the reduced treatment time for the accurately diagnosed group.

## 5. Conclusion

Though slightly less sensitive, the subtype-determining questionnaire still bears a competent screening power in identifying patients with BPPV in the geriatric population. It is also capable of eliciting valuable referable information to facilitate an efficient diagnosis.

## Data availability statement

The raw data supporting the conclusions of this article will be made available by the authors, without undue reservation.

## Ethics statement

The studies involving human participants were reviewed and approved by the Ethics Committee of Peking University International Hospital. Written informed consent for participation was not required for this study in accordance with the national legislation and the institutional requirements.

## Author contributions

YW designed the study, collected clinical data, and wrote the manuscript. YL performed the positional tests and treatment of the patients. JS supervised and guided the study. All authors contributed to the article and approved the submitted version.

## Footnotes

1
R-project.org/
